# Impact of restoring male fertility with transplantation of in vitro propagated spermatogonial stem cells on the health of their offspring throughout life

**DOI:** 10.1002/ctm2.531

**Published:** 2021-10-12

**Authors:** Joana B. Serrano, Rik van Eekelen, Cindy M. de Winter‐Korver, Saskia K.M. van Daalen, Nils C. Tabeling, Lisa A.E. Catsburg, Marion J.J. Gijbels, Callista L. Mulder, Ans M.M. van Pelt

**Affiliations:** ^1^ Reproductive Biology Laboratory, Center for Reproductive Medicine, Amsterdam UMC, Amsterdam Reproduction and Development Research Institute University of Amsterdam Amsterdam The Netherlands; ^2^ Center for Reproductive Medicine, Amsterdam UMC, Amsterdam Reproduction and Development Research Institute University of Amsterdam Amsterdam The Netherlands; ^3^ Department of Medical Biochemistry, Experimental Vascular Biology, Amsterdam UMC University of Amsterdam Amsterdam The Netherlands; ^4^ Department of Pathology CARIM, Cardiovascular Research Institute Maastricht, GROW‐School for Oncology and Developmental Biology Maastricht University Maastricht The Netherlands

Dear Editor,

Spermatogonial stem cell transplantation (SSCT) is a potential novel fertility treatment for prepubertal cancer patients that require gonadotoxic treatment. It comprises in vitro propagation of SSCs from a cryopreserved testis biopsy followed by autotransplantation into the infertile patient's adult testis resulting in full recovery of spermatogenesis. In this blinded preclinical study, the long‐term health of SSCT‐derived offspring was assessed for the first time, using a systematic blueprint testing throughout life. No major differences in health outcomes between mice born after SSCT in two consecutive generations (first generation [F1] and second generation [F2]) and control were found, thereby providing crucial evidence that SSCT is a safe procedure.

Due to its germline character, SSCT is the only adult stem cell‐based transplantation therapy that can restore male fertility and may not only affect the recipient, but also the offspring. Remarkably, medically assisted reproductive (MAR) therapies have been introduced in the clinic after preclinical studies focusing mainly on its efficacy instead of safety for the offspring.^1^, ^2^ Unfortunately, after clinical implementation, children born after some of these MARs have increased risk for congenital abnormalities and low birthweight,^3^, ^4^, ^5^, ^6^ developmental delays and cardiometabolic disease later in life.^3^, ^4^, ^7^ To ensure the health of future generations born from fathers that have received SSCT to restore fertility, we here studied indicators of health in the offspring throughout life that can be linked to potential risk factors of developmental problems, cardiometabolic disease, and ultimately result in increased mortality and pathologies^2^ (Supporting Information).

Birth assessments showed that congenital abnormalities were a rare event (control *n* = 1/151, SSCT‐F1 *n* = 3/125, SSCT‐F2 *n* = 4/123; Figure 2A) with no statistically significant differences found in either SSCT generation compared to control (odds ratio [OR]: 3.78, 95% confidence interval [CI]: [0.14, 312.36]) for F1 and (OR: 4.18, 95% CI: [0.60, 82.90]) for F2 (Figure 2A), although the estimated odds ratios were relatively high. Among these abnormalities, spinal defects were most common among all groups, including control (Figure 2B,C). Birthweight and birth length (Figure 2D) were similar between control and SSCT offspring, in both generations. Despite performing appropriate power calculation, based on the known incidence of congenital abnormalities in other MARs, the estimated sample size (*n* = 116 per group) may have been a limitation in determining the effect of SSCT on congenital abnormalities in the offspring. This was probably because congenital abnormalities were rare, which is clearly shown in the broad confidence intervals.^8^

**FIGURE 1:**
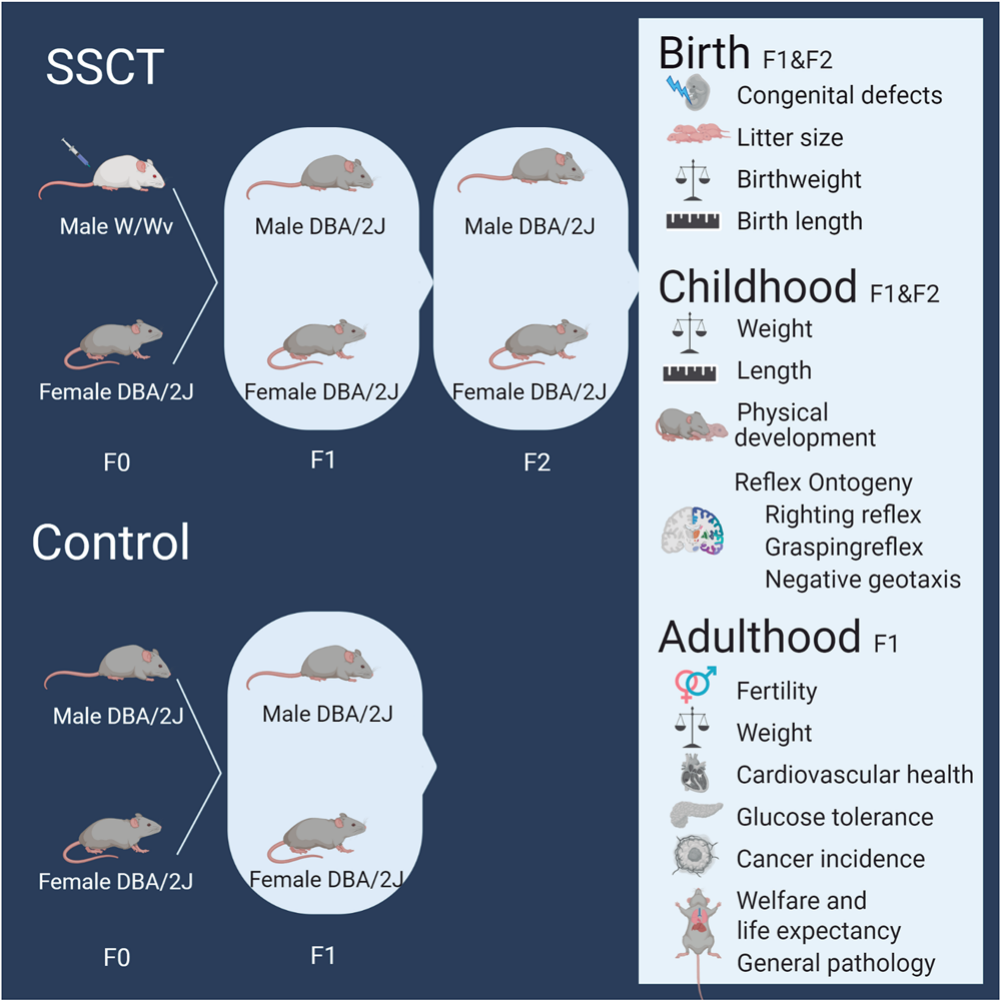
Study design to determine development and health of spermatogonial stem cell transplantation (SSCT) offspring compared to control offspring. Sterile W/Wv male mice received SSCT to restore fertility. Control and transplanted males were placed in breeding with control females. The control group was bred for one generation for welfare reasons, while the SSCT group was bred for two generations to determine if any developmental or health deviations at birth, childhood, and adulthood are inherited. F0: parental generation, F1: first generation, F2: second generation

**FIGURE 2:**
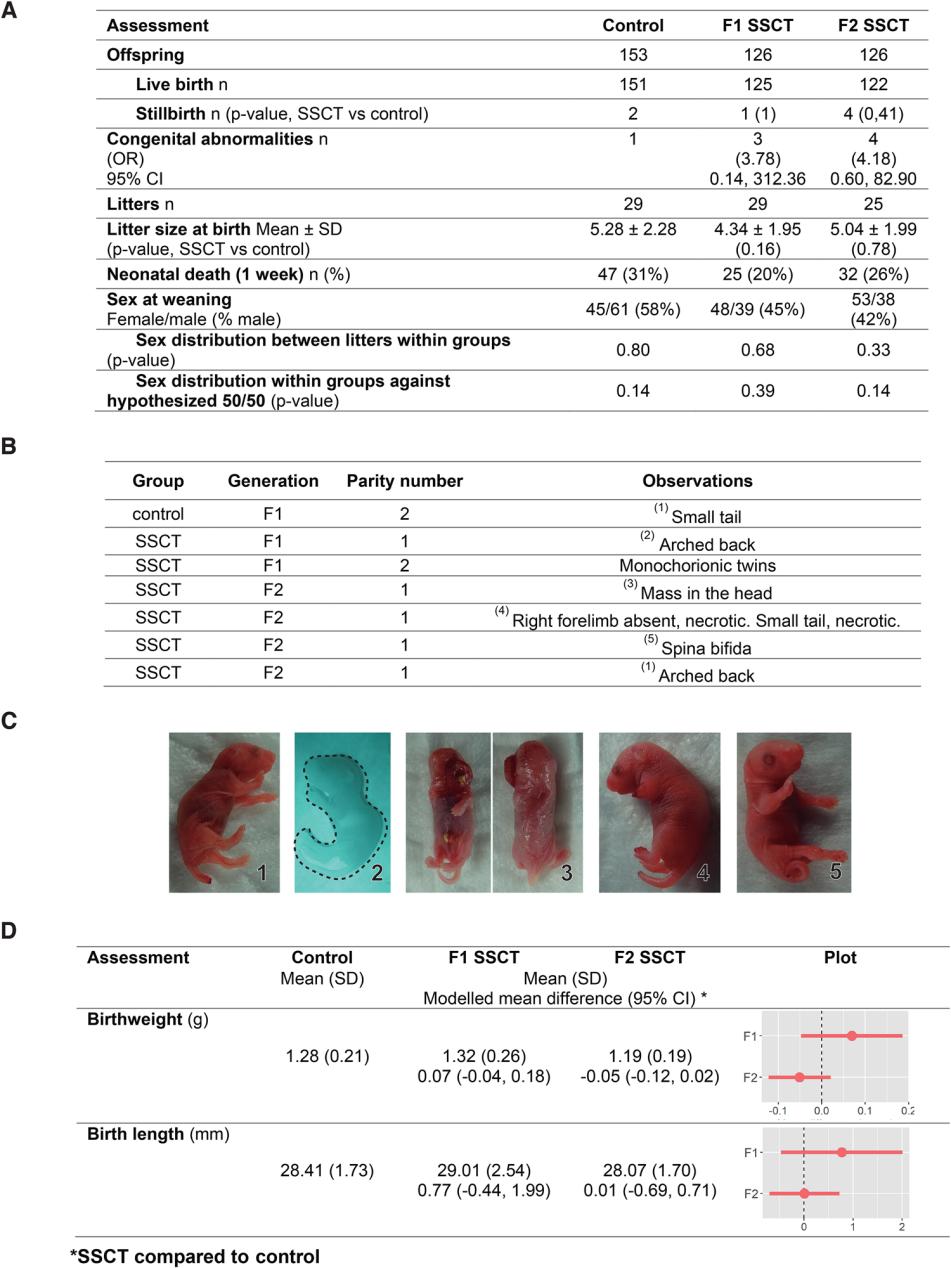
Birth characteristics and analysis of spermatogonial stem cell transplantation (SSCT) compared to control. (A) Birth hallmarks: stillbirth (Fisher's exact test), sex distribution (Fisher's exact test, exact binomial test), and litter size (Mann‐Whitney‐Wilcoxon rank‐sum test) of offspring of different treatment groups. *p*‐value < 0.05 (SSCT compared with the control group). (B) Distribution of congenital abnormalities in control and SSCT offspring, in both generations: first generation (F1) and second generation (F2). (C) Photographs of congenital abnormalities. (D) Birthweight and birth length are similar in first‐ and second‐generation control and SSCT offspring. Indicated as mean (SD) and modeled mean difference (mixed models) control versus SSCT 95% CI, and graphical representation of modeled means ±SE

During the childhood period (first 28 days of life), the timing of physical and neurodevelopment was estimated by the occurrence of essential morphological landmarks and reflexes.^1^, ^2^ No differences were found between control and SSCT‐derived offspring, in both generations, for fur growth, the timing of eye and ear opening, lower incisor eruption, and development of correct righting (Videos S1–3), grasping (Videos S4,5), negative geotaxis (Videos S6–S8) reflexes (Table S1 and Figure 3) and length (Figure S1). However, a statistically significant difference of 1‐day earlier upper incisor eruption (95% CI: [–2.09, –0.14]) and decreased weight gain (95% CI: [–0.42, –0.08]) were observed between SSCT‐F2 and control, but not in F1 (Figure 3).

**FIGURE 3:**
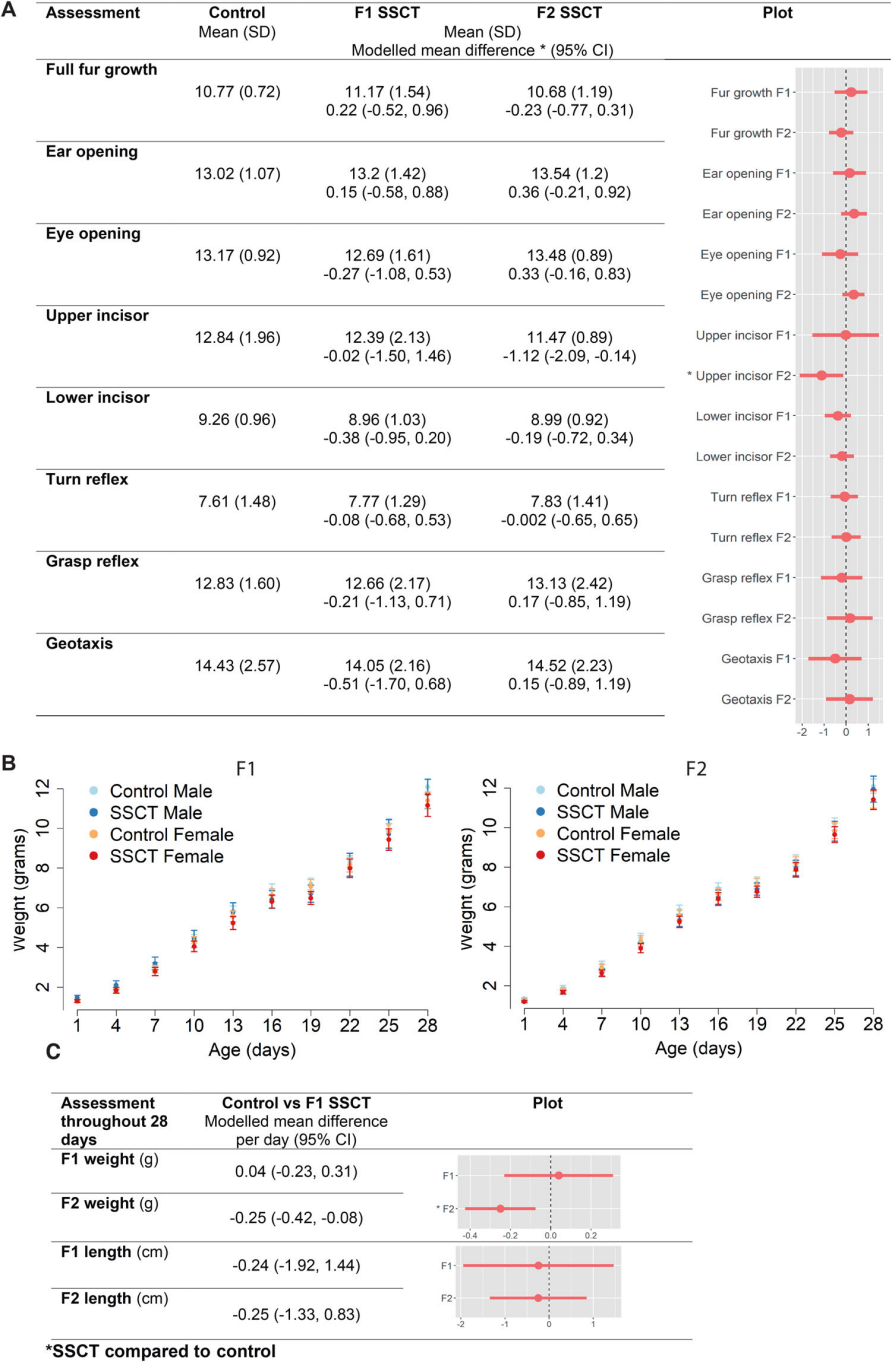
SSCT childhood development and health compared to control. (A) The day that physical developmental milestones and behavioral reflexes were reached. Spermatogonial stem cell transplantation (SSCT) has minimal effect on physical developmental and behavioral reflexes. (B) Graphical representation of weight during the first 28 days of life. (C) Weight and length during the first 28 days indicated as mean (SD) and modeled mean difference (mixed models) control versus SSCT 95% confidence interval (CI)

During adulthood, cardiometabolic risk factors and fertility were assessed.^3^, ^7^, ^8^ Weight was consistent between control and SSCT offspring, suggesting no risk of obesity in both generations (F1 at 18 months 95% CI: [–1.29, 1.1] and F2 at 6 months 95% CI: [–0.44, 1.33]; Figure 4A and Figure S2, respectively). Although, the weight of the SSCT offspring in the F2 was significantly decreased compared to control during childhood, reassuringly, after reaching adulthood, F1‐control and SSCT‐F1/F2 offspring presented similar weight curves through life, indicating recovery from the reduced weight found during childhood in the F2. Additionally, all F1 animals used as breeding pairs were fertile. Furthermore, to assess glucose intolerance as a risk for diabetes, we first determined weight and fasting glucose levels (Table S1) and found no statistically significant differences between SSCT offspring and control (Figure 4B). Glucose intolerance was tested after glucose injection also revealed no differences between SSCT and control offspring (Figure 4B). Cardiovascular assessments, including diastolic blood pressure, systolic blood pressure and heart rate showed no differences between control and SSCT‐F1 (Figure 4C), indicating no risk for cardiovascular disease.

**FIGURE 4:**
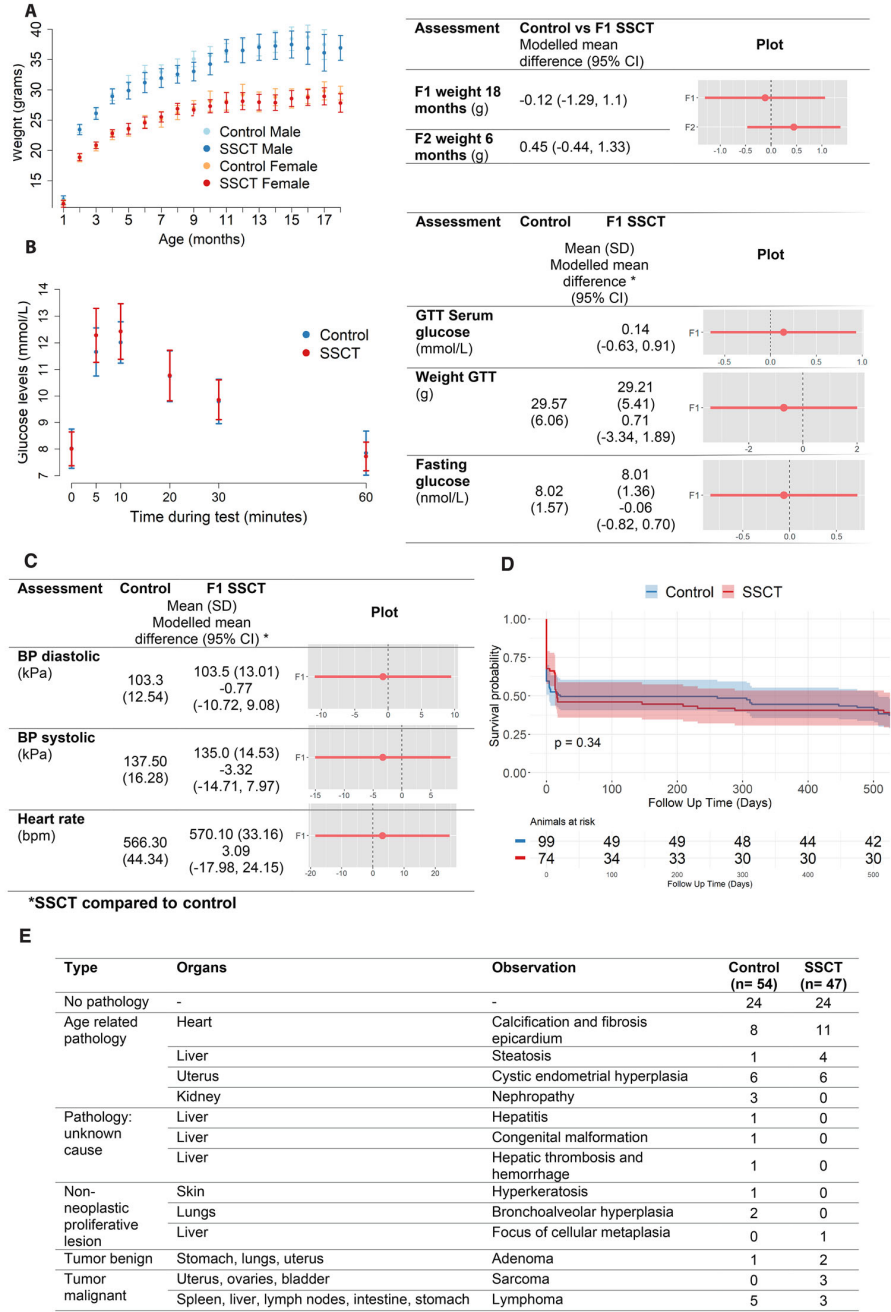
Adult growth and health in spermatogonial stem cell transplantation (SSCT) compared to control. Adult growth, life expectancy, and pathologies in SSCT compared to control. (A) Graphical representation of the first‐generation (F1) control and SSCT weight for 18 months. (B) Metabolic assessment measured by weight and glucose tolerance test (GTT) in F1‐control and SSCT mice at 11 months of age. The offspring was weighed before GTT and fasting glucose levels were taken. (C) Cardiovascular assessment was measured by heart rate, systolic and diastolic blood pressures at 9–11 months of age. Mean (SD) and modeled differences (mixed models) control versus SSCT 95% CI, and graphical representation of modeled mean ± SE. (D) Time‐to‐death Kaplan–Meier curve in days showing no differences between SSCT‐F1 and control in life expectancy (log‐rank test). (E) Frequency of organ pathology at 18 months of age. Only the most severe pathology is presented in cases with multiple pathologies

Ultimately, we analyzed the mortality rate and general pathology of the animals. Overall, the life expectancy of the offspring up until the end of the study (18 months) was similar between control and SSCT, registering a similar incidence of premature death throughout life (Figure 4D and Table S2). Histological abnormalities were overall comparable between control and SSCT comprising calcification in the epicardium, steatosis, cystic endometrial hyperplasia, and nephropathy (Figure 4E, only the most severe pathology is reported). Most pathologies were associated with aging or strain‐specific (DBA/2J) complications.^9^ Overall, tumor incidence was similar between both groups and characteristic of DBA/2J, including adenomas (control = 1, SSCT = 2) and lymphomas (control = 5, SSCT = 3). In the SSCT group, three animals developed sarcoma against none in the control group, which may be a coincidental finding and needs further research. In future clinical trials, the occurrence of sarcomas should be monitored in detail. Regarding the weight of the organs, a significant increase in the weight of the testes, male genital fat pads, and pancreas in SSCT and decreased weight for the right ovary, liver, and stomach (Figure S3) was found, with no corresponding phenotype. Except for testicular histology, with confirmed fertility by the occurrence of spermatogenesis in all the F1 offspring, showing statistically significant larger mean tubular diameter of round seminiferous tubules SSCT (192 ± 21.3 μm) compared to control (159 ± 19.8 μm, *p* = 0.004; Figure S4). Together with fewer germ cells inside control tubules, this may suggest a reduced aging effect in SSCT offspring.

In conclusion, finding no major impact of SSCT on multiple markers of development and general health of two consecutive generations through life, including birth characteristics, childhood development, and adult health, this preclinical study is an important step toward clinical translation of SSCT for infertile childhood cancer survivors. Together with a previous safety and efficacy study on SSCT‐recipient health,^10^ this study provides crucial evidence for requesting ethical approval of SSCT for a phase‐1 clinical trial, which should include thorough follow‐up of the transplanted men and their children.

## CONFLICT OF INTEREST

All authors declare they have no conflict of interests.

## FUNDING INFORMATION

Stichting Kinderen Kankervrij (Dutch Children Cancer‐Free Foundation); Grant/Award number: KiKa#86 (Ans M.M. van Pelt) ZonMW TAS; Grant/Award number: 116003002 (Ans M.M. van Pelt); Amsterdam UMC, University of Amsterdam.

## AUTHOR CONTRIBUTIONS

Conceptualization and methodology: Ans M.M. van Pelt, Joana B. Serrano, and Callista L. Mulder. Software and formal analysis: Rik van Eekelen and Joana B. Serrano. Validation: Joana B. Serrano. Investigation and writing‐review and editing: Joana B. Serrano, Callista L. Mulder, Ans M.M. van Pelt, Cindy M. de Winter‐Korver, Saskia K.M. van Daalen, Nils C. Tabeling, Lisa A.E. Catsburg, and Marion J.J. Gijbels. Writing‐original draft: Joana B. Serrano, Callista L. Mulder, and Ans M.M. van Pelt. Visualization: Joana B. Serrano. Supervision: Ans M.M. van Pelt and Callista L. Mulder. Project administration: Joana B. Serrano and Cindy M. de Winter‐Korver. Funding acquisition: Ans M.M. van Pelt.

## DATA AVAILABILITY STATEMENT

All data, code, and materials are available upon request from the corresponding author.

## Supporting information

SUPPORTING INFORMATION
