# Supplementary material for: Impact of restoring male fertility with transplantation of in vitro propagated spermatogonial stem cells on the health of their offspring throughout life
Source: Clin Transl Med. 2021 Oct 12;11(10):e531. doi: 10.1002/ctm2.531 (PMC8506643; doi:10.1002/ctm2.531)
Supplement: Supplementary file 1 — SUPPORTING INFORMATION [file CTM2-11-e531-s001.docx]

**Supplementary Materials**

Impact of restoring male fertility with transplantation of in vitro propagated spermatogonial stem cells on the health of their offspring throughout life

Joana B. Serrano, Rik van Eekelen, Cindy M. de Winter-Korver, Saskia K.M. van Daalen, Nils C. Tabeling, Lisa A.E. Catsburg, Marion J.J. Gijbels, Callista L. Mulder1*‡, Ans M.M. van Pelt1*‡

*Corresponding author. Emails: [a.m.vanpelt@amsterdamumc.nl](mailto:a.m.vanpelt@amsterdamumc.nl) and [c.l.mulder@amsterdamumc.nl](mailto:c.l.mulder@amsterdamumc.nl)

‡These authors contributed equally to this work as co-senior authors.

**This PDF file includes:**

Methods

Figs. S1 to S4

Tables S1 to S4

Legends for movies S1 to S8

Supplementary references

**Other Supplementary Materials for this manuscript include the following:**

Movies S1 to S8

**Methods**

**Mice Husbandry**

All mice were maintained on a 14:10h reversed day-night cycle, with standard rodent chow diet (Envigo 2016 Teklad global 16% protein rodent diet) and water available ad libitum in open cages. The animals were housed socially with their littermates of the same sex and the placing of the cages was changed periodically throughout the room. Trained researchers and animal caretakers were blinded for the origin of the animals throughout the entire study period while recording all observations in an experimental logbook that registers the handling of each animal. When required, a veterinarian or animal welfare officer was contacted for advice on welfare issues. An independent researcher was responsible for administrative monitoring throughout the preclinical study: arranging breeding schemes; selecting the animals if required; documenting all the animals born and dead; and kept the key for the blinded researchers. When choosing animals for the different health assessments or breeding schemes, animals derived from the same parent couple were avoided to circumvent parental effects.

**Mice and study design**

To study the health SSCT-derived offspring, mimicking future human SSCT (Figure 1), SSCs were isolated from cryopreserved testis of neonatal DBA/2J mice (Charles Rivers), propagated *in vitro* for at least 50 days and transplanted to colonize the testis as previously described [1, 2]. To assure the offspring is originated from cultured SSCs, sterile males were used as recipients for transplantation at 6 weeks of age (W/W-v, Jackson Laboratories). SSCT males were placed with wild-type DBA/2J females (Charles Rivers) to produce two generations of offspring. As a parallel control group for SSCT-F1 and SSCT-F2, wild-type DBA/2J females and males (Charles Rivers) were placed in breeding to generate one generation of offspring.

To test the health of offspring generated via this novel MAR, we used an array of tests [3] in a multi-generational mouse model (Figure 1). At birth, the pups were checked for congenital abnormalities, birthweight and length. During the first 28 days, individual animals were checked periodically for physical developmental milestones and behavioral reflexes. As the mice reached adulthood, fertility was assessed by the ability to reproduce. Furthermore, cardiometabolic health was analyzed: animals were divided into groups to test glucose tolerance, blood pressure, heart rate and obesity. The welfare and life expectancy of the animals were followed up to 18 months of age. Post-mortem assessment was performed after sacrificing at 18 months to check for the impact of SSCT on general pathology.

**Study approval**

All animal experiments were licensed by the Dutch competent authority (License no AVD1180020171524) according to the European Directive 2010/63/EU on the protection of animals used for scientific purposes. The experiments and competence of the personnel were overseen by the Animal Welfare Body of the Amsterdam UMC, location Academic Medical Center.

**Neonatal testis cryopreservation**

Murine DBA2/J neonatal testes tissue was cryopreserved (4-8 days post-partum) for culture and subsequent transplantation. First, male pups were identified by anogenital distance and sacrificed with C02/O2 followed by C02 asphyxiation and finally decapitation. Using a dissection microscope, the testes were located, taken out and the epididymis and tunica vaginalis were carefully removed. Multiple testes were pooled and cryopreserved in a cryovial with MEM (Minimum Essential Medium, Thermo Fisher Scientific) with 20% FCS (Fetal Calf Serum, Thermo Fisher Scientific) and 8% DMSO (Dimethyl sulfoxide) in a Coolcell® freezing canister and finally stored in vapor phase liquid nitrogen.

**SSCs culture**

Based on Shinohara’s original protocol, the testis tissue was washed in HBSS (Gibco) and digested with 1-2 mL of collagenase (1 mg/mL, 125 units/mg in CMF-HBSS with DNase) followed by incubation at 37°C for 15 minutes (shaking on 120 cycles/min). After washing again with HBSS the tissue was incubated in 0.25% Trypsin/EDTA (Gibco), HBSS and DNase at 37°C, while shaking for 10 min. After inactivation with MEM10%FCS and centrifugation, the cells were plated in a 0.1% gelatin coated plates at a density of 200.000 cells/well in plated in supplemented Stem Pro-34, as previously described [4]. The next day, floating cells were passaged in non-coated plates. The first passaging occurs after 10-14 days by trypsinization, the second passaging after 20-28 days, and the third passaging from 30-38 days. From the third passage, cells were transferred to Mitomycin C (Sigma) inactivated MEFs, which were renewed every 2 weeks by differential plating for 60 min on gelatin coated plates as previously described [5]. Ultimately, the cells were cultured up to 50 days to at least passage 5 (for transplantation).

**SSCs cryopreservation, thawing and preparation**

For harvesting SSCs, the cells were washed with PBS, trypsinized, and then plated in 0.1% gelatin coated plated and MEM10%FCS, for 60 minutes so that somatic cells including the MEFs attach, while other cells will keep on floating and are cryopreserve in 8% DMSO after centrifugation.

Before the transplantation procedure, frozen cells were thawed rapidly with warm tap water until the ice clump is loose from the walls. The cells were then washed with pre-warmed HBSS, counted, and resuspended in 10µl of HBSS before transplantation. Overall, 0.04-0.5 million SSCs were injected into each recipient testis. Right before transplantation, trypan blue was added to the cell suspension (1:5, so 2.5µl to the 10 µl suspension).

**SSC transplantation**

As previously described [3], recipient W/W-v mice we anesthetized with 2-3% isoflurane via inhalation. The procedure is as follows: the abdomen skin wall was then lifted with forceps and an incision was made with surgical scissors. Carefully, the peritoneal cavity was opened, and forceps were uses to hold the skin wall and search the fat pads attached to the testis in the cavity, to exteriorize the testis. The efferent ducts were identified and prepared. Cells were transplanted into the seminiferous tubules of the testis through the efferent ducts using a capillary with a syringe and a custom glass capillary. The injection was performed into the efferent ducts in the direction of the rete testis and ceased when there is capillary tension and surface seminiferous tubules were filled by the Trypan blue dye. The transplantation was performed bilaterally. Animals received Temgesic (0.05 mg/kg, Reckitt Benckiser Healthcare, subcutaneously) as an analgesic prior and 1-2 days after the transplantation. Finally, the recipient received immunosuppression using 0.50 µg anti-CD4 via intraperitoneal injection (eBioscience, Clone GK1.5) on days 0, 2 and 4 following the transplantation.

**Neonatal and post-natal development**

Three to five months after transplantation both control and SSCT males were placed in breeding. When a pregnant female was identified, she was isolated to a new cage in order to give birth without accompanying animals. Birth was checked daily.

All animal experiments were performed during the dark period, using red light in the room and recorded with a night vision camera (Sony FDRAX53/B 4K HD Night Shot Camcorder). At birth, the litter size, occurrence of birth defects, birthweight and length of pups were documented, and the animals were photographed. Pups received an identifying intradermal tattoo on the day of birth.

During the first 28 days multiple tests were performed according to their specific periods of development as described earlier [3]: congenital abnormalities, ear opening, eye opening, fur growth, incisor eruption were checked, along with behavioral reflex testing of negative geotaxis, grasp and righting reflexes (Figure 1). Offspring were weighed and length was measured on the day of birth and subsequently every 3 days, for 28 days. Pups were sexed at weaning (day 28) by means of the anogenital distance, which is longer in males [6].

In wild-type naturally conceived animals, the mouse is born naked with closed ears and eyes. Between days 2-4 hair starts to appear, around day 12 incisor grow out, between day 13-14 the ears open, and between days 14-15 the eyes also open [7, 8]. Around day 16, when the eyes are completely functional, the pups will begin to eat solid food but nursing can continue for one more week. After three weeks of age the pups resemble an adult mouse except for their size and differentiation of the sexual organs [9].

During the first three weeks several aspects of physical development were monitored according to a modified protocol of the morphological screening developed by van der Meer et al. [10]. The weight and crown to tail length were measured at intervals of 3 days (day 1, 4, 7 etc.). At precise time intervals [3, 10] we registered the day of hair growth (day 4-14), opening of the eyes (day 10-17), opening of the ears (day 10-17), and tooth development (day 8-13). The tests were evaluated based in incremental developmental levels for each characteristic as described earlier [3]. Briefly, for hair growth, no hair present corresponds to a level 0, when the fur starts as fine stubble over the back it is level 1, a complete coat of fine fuzzy fur it is a level 2, and totally covered with thick hair it is a level 3. For lower and upper incisor eruption when the incisors are visible, but not erupted it is a level 1 and in level 2 the incisors are fully erupted. While testing eye opening, in level 1 the eyelids start to open, slit-like palpebral opening, and in level 3 the eyelids are totally open. In ear opening tests, level 1 is when the ears start to open, slit-like opening, and level 2 is when the ears are completely open.

Reflex ontogeny protocols were also adapted from van der Meer et al. [10]. We included the surface righting reflex from days 4 to 13 as previously described [3], which is the ability of regaining the normal position after the mouse pup is placed on its back. The ideal response is when the animal rights itself immediately demonstrating labyrinthine reflexes and complex coordinated action involving muscles in the neck, trunk, and limbs. In starting level 1 (video 1) the animal lies on its back, moving its paws in the air without any direction and there is no righting. When the animal rights itself, but slowly, it is a level 2 (video S2), and finally in level 3 the animal rights itself immediately and stands on its four paws (video S3).

Grasping reflex was performed between days 8-21 as previously described [3] to evaluate fine motor skills of the mice. The wooden end of a cotton bud is placed in the inside of the forepaw of the pup and the mature response occurs when the animal immediately grasps it. In starting level 1 (video S4) the animal puts its paw on the cotton bud, but it does not hold, while in level 2 (video S5) the object is firmly grasped and the mouse it is able to grasp and hold the stick.

Negative geotaxis assesses motor coordination and labyrinthine reflexes in young pups from day 9 to 21 as previously described [3]. The mice are placed in a 45° slope facing downwards and the mature response is to immediately rotate 180° to the head-up position and starts to climb up the slope. A delayed or failed response to turn upwards could indicate deficits in coordination, balance, or vestibular input. In starting level 1 (video S6) is when the animal turns its body slowly on the slope, but stops halfway without moving any further, in level 2 (video S7) the animal turns its body, and then goes up the ramp but stops halfway, while finally, in level 3 (video S8) the animal turns by 180° and moves towards the top of the slope immediately reaching the top.

The number of animals analyzed in each test is available in Table S3.

**Cardiovascular and metabolic analysis**

For blood pressure and glucose tolerance test (GTT), animals from different parents were selected by an independent researcher according to the samples size calculated (Table S4). The protocols were followed with the corresponding guidelines and procedures [11–13], together with advice from experts within in our institute.

To determine the cardiovascular health of the offspring, systolic blood pressure (SBP), diastolic blood pressure (DBP) and heart rate was determined at 9 months old by tail-cuff method (CODA Surgical Monitor, Kent Scientific, Torrington, CT) which measures changes in tail blood volume. DBA/2J F1 mice were acclimatized to the tail cuff and heating pad apparatus for 4 days and several minutes before data collection, every time the readings were taken. The occlusion cuff was placed at the base of the tail, and the volume-pressure recording cuff was placed below the occlusion cuff; the cuffs then began the inflation/deflation cycles. Ten recordings were taken per mouse at the fifth day for the final cardiovascular testing.

In order to assess diabetic phenotypes in the offspring a GTT was performed in first-generation SSCT and control DBA/2J female and male offspring at 8-10 months of age (sample size from power calculation in Table 4). The weight of the selected animals was taken the day prior, as it can influence GTT [14]. The animals were fasted (only drinking water) for 4h prior to testing to establish a stable baseline measurement. GTT was performed directly after the light period. Animals were challenged with an intraperitoneal injection of glucose (1gram/ml/kg). Blood samples were obtained from the tail tip for assessment of glucose at 0, 5, 10, 20, 30 and 60 min after glucose injection with a glucometer (Accu-Chek Performa; Roche Diagnostics) before sacrificing the animals with induced by C02/O2 followed by C02 asphyxiation and finally cervical dislocation after the final measurement.

To determine whether control or SSCT weight developed differently at offspring were weighed monthly for the duration of the experiment until 18 months of age. The number of animals analyzed in each test is available in Table S3.

**General pathologic assessment**

Necropsy, if possible, was performed if animals became ill or died suddenly. All remaining animals were sacrificed at 18 months under total anesthesia induced by CO2/O2, followed by CO2 asphyxiation and finally cervical dislocation. General health and cancer incidence were studied by evaluating the organs of the animals macroscopically and registering all abnormalities. Overall, the organs were collected and fixated in 4% PFA and stored in ethanol at 4°C for histology. The following organs were weighed and photographed upon removal: testis, epididymis, uterus, ovary, urine bladder, spleen, intestine, stomach, liver, kidney, lungs, heart, thymus and brain. Findings were reported in a necropsy report. In case an animal was found dead, time of death was estimated. Necropsies were performed mostly by one researcher.

The organs of interest (i.e. testis and abnormal organs) were embedded in paraffin for histological examination after HE staining. The resulting slides were analyzed using an Olympus BX41 bright-field microscope with an Olympus DP20 color camera (Olympus, Shinjuku, Tokyo, Japan). Histological abnormalities were confirmed by a blinded pathologist and the results were presented for the most severe pathology found in each animal.

Histology of the testis of F1 control and SSCT mice was performed to assess the presence of ongoing spermatogenesis. The size of the seminiferous tubules was examined by a researcher who performed the evaluation blinded for the origin of the testicular tissue. The average size of the shortest diameter of round tubules was compared between groups. Only seminiferous tubules that had the smallest diameter longer than 75% of the biggest diameter of the tubule were considered.

**Power calculation**

Sample size and power calculations were performed in Stata (IC 14.2): one-sided, with continuity (Table S4). Our primary outcome was the presence of congenital abnormalities in SSCT offspring. Therefore, we hypothesized that SSCT offspring were more likely to develop congenital abnormalities than control animals with the general reported incidence of 10–15% [15–17]. Based on these estimations, we calculated that at least 116 animals for each generation would be needed to detect a difference of 15% in congenital abnormalities with a power of 80% with an α of 0.05. Individual power calculations were performed for the secondary outcomes birthweight, righting reflex, glucose tolerance test (GTT) and blood pressure (Table 4). No correction for multiple testing was performed as a conservative option, given the study was on safety [18].

**Statistical Analysis**

Statistical analysis was performed using multilevel mixed effects linear model in R with the lmer() and glmer() functions in the lme4 package (R software, version 3.6.2, R Project for Statistical Computing). These models take into account the hierarchical nature of the datasets, most notably clustering within litters, and different fixed and random effects [19] (group - SSCT versus control, sex, parent ID - the unique combination of father and mother, body weight, parity number - the fact that the animals belong to the 1st up to 4th litter). Congenital abnormalities were analyzed with mixed effects logistic regression (fixed effects: group, parity; random intercept: parent ID), and the calculated odds ratios using Firth's correction for logistic regression yielded similar results. Birthweight, birth length, fur growth, ear and eye opening, upper and lower incisor, righting grasp and negative geotaxis reflexes were analyzed by using mixed effects linear regression (fixed effects: group, parity, sex; random intercept: parent ID). Longitudinal analysis of the weight throughout the first 28 days, 18 months (F1) or 6 months (F2) was performed with a mixed effects linear regression (fixed effects: group, parity, sex; random intercept: parent ID, time). The weight of the organs measured during necropsy was analyzed by using the mixed effects linear regression (fixed effects: group, parity, body weight, and sex – except for ovaries, uterus, fatpads, testes and epididymides where sex could not be a fixed effect; random intercept: parent ID). One animal with a sarcoma was removed from the analysis of organ’s weights as her organs were abnormally large and this had a large influence the mean weight in the results. To evaluate the differences between groups, the coefficients for the group comparison and their respective 95% confidence intervals (95% CI) based on profile likelihood are presented (Figure 2-4).

Litter size was analyzed with Mann-Whitney-Wilcoxon rank sum test with continuity correction in R with the wilcox.test() function. Sex distribution within litters of each experimental group was analyzed with the Fisher's exact test in R with the fisher.test() function. Sex distribution of each experimental group compared to expected null hypothesis of 50/50 was analyzed with the exact binomial test in R with the binom.test() function. Stillbirths were compared with a Fisher's exact test in R with the fisher.test() function. Testicular diameter differences between the seminiferous tubules of both groups were analyzed by Student’s t-test in Microsoft Excel. Log-rank test was used to compare cumulative survival curves between the two groups with the survdiff() function in R, throughout the length of the study. Animals that were sacrificed for other assessments (i.e. GTT) were excluded from survival analysis. The number of animals analyzed in each test is available in Table S3.


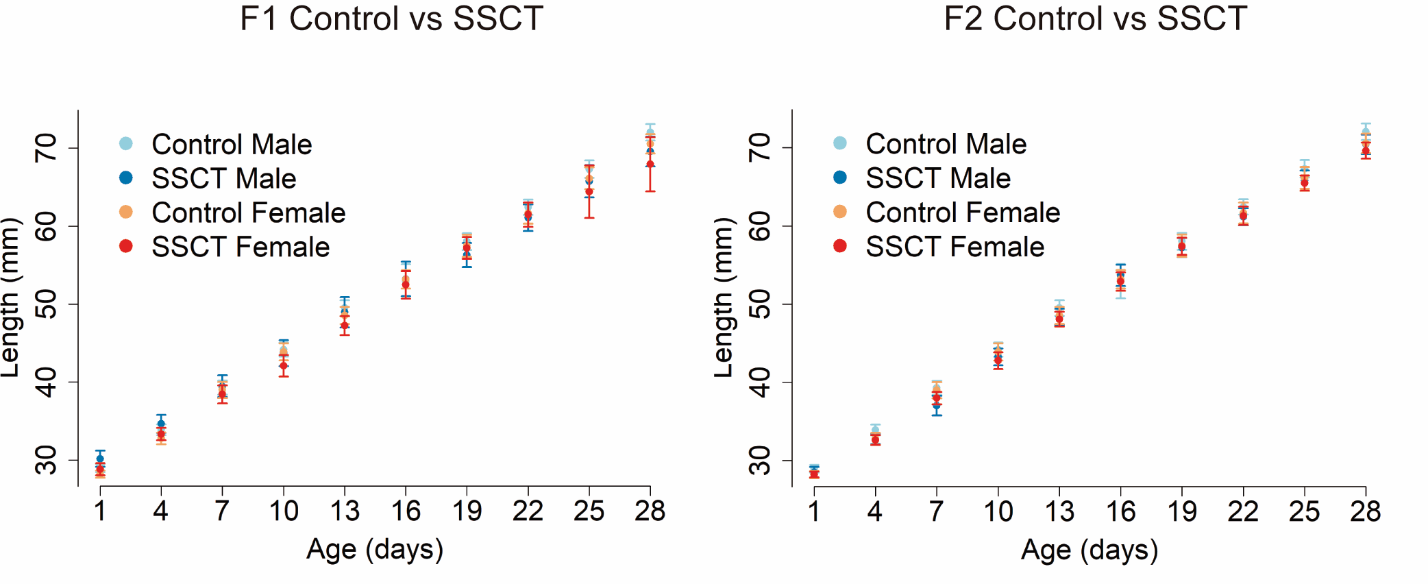
**Fig. S1. SSCT childhood development and health compared to control.** Graphical representation of length during the first 28 days of life.

**
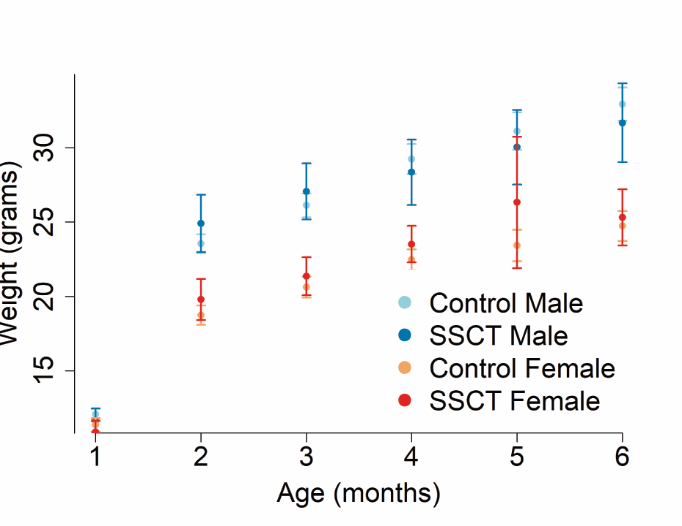
**

**Fig. S2. Adult weight in F2 SSCT compared to F1 control. (A)** Graphical representation of the F1 control and F2 SSCT weight for 6 months.

| Weight (mg) | Control | F1 SSCT | Plot |
| --- | --- | --- | --- |
|  | Mean (SD)  Modelled mean difference (95% CI) * | |  |
| Ovary R | 26.04 (7.54) | 19.96 (5.27)  -5.18  (-9.09, -1.26) ^a^ | 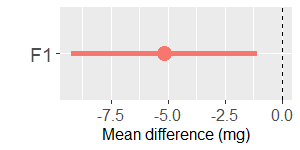 |
| Ovary L | 39.26 (53.24) | 21.63 (5.48)  -15.95  (-36.77, 4.88) | 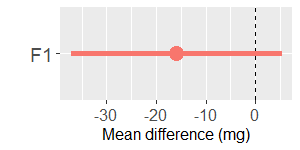 |
| Uterus | 307.60 (112.57) | 319.40 (81.64)  5.01  (-50.30, 63.68) | 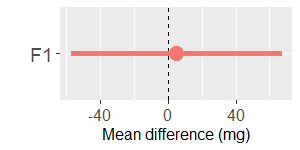 |
| Fat pad R female | 925.90 (345.46) | 706.50 (399.31)  0.35  (-144.84, 146.43) | 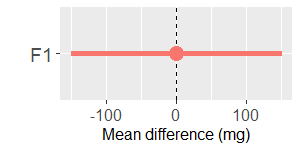 |
| Fat pad R male | 607.40 (240.40) | 839.20 (381.01)  306.89  (207.63, 406.16) ^a^ | 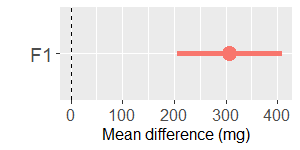 |
| Fat pad L female | 834.30 (301.23) | 681.70 (381.01)  23.19  (-127.03, 173.67) | 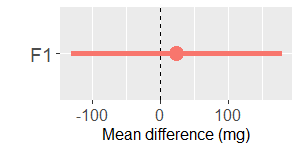 |
| Fat pad L male | 554.60 (222.45) | 782.10 (355.92)  263.08  (167.50, 358.66) ^a^ | 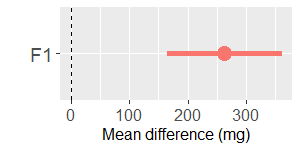 |
| Testis R | 81.21 (22.03) | 101.50 (22.79)  20.63  (8.38, 33.52) ^a^ | 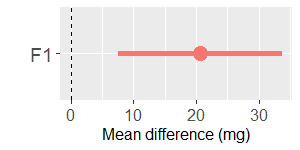 |
| Testis L | 72.63 (17.57) | 96.85 (18.76)  23.93  (14.05, 34.35) ^a^ | 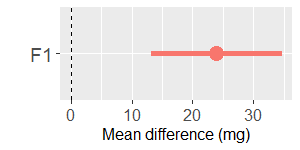 |
| Epididymis R | 21.72 (7.81) | 26.97 (6.77)  4.08  (-1.74, 9.88) | 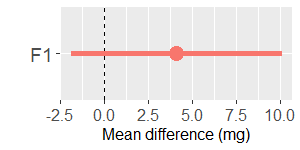 |
| Epididymis L | 22.19 (6.34) | 24.05 (3.81)  2.39  (-1.05, 5.84) | 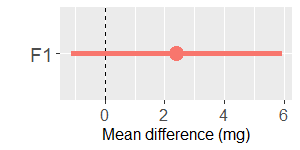 |
| Bladder | 25.08 (5.10) | 23.30 (4.90)  -1.64  (-3.77, 0.47) | 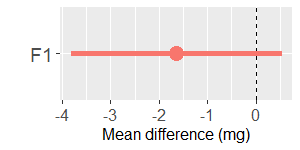 |
| Pancreas | 407.60 (53.75) | 431.30 (105.57)  35.89  (4.38, 67.40) ^a^ | 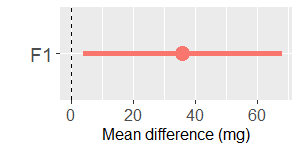 |
| Intestines | 1179.00 (158.01) | 1115.00 (150.81)  -62.09  (-138.61, 13.61) | 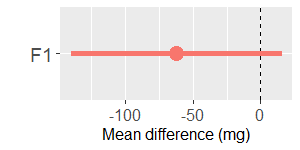 |
| Spleen | 180.40 (336.76) | 162.13 (382.29)  -17.49  (-166.41, 131.43) | 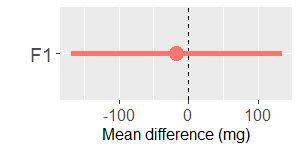 |
| Stomach | 287.00 (57.07) | 248.60 (37.72)  -24.91  (-40.94, -8.89) ^a^ | 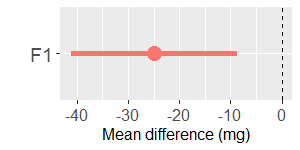 |
| Liver | 1939.00 (644.67) | 1600.00 (432.11)  -182.64  (-344.10, -21.17) ^a^ | 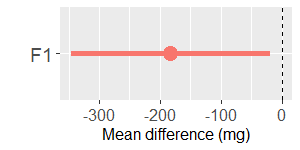 |
| Kidney R | 256.30 (77.60) | 224.60 (71.34)  -4.46  (-17.16, 8.24) | 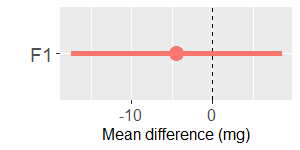 |
| Kidney L | 252.60 (74.63) | 224.30 (73.48)  -2.14  (-16.00, 11.73) | 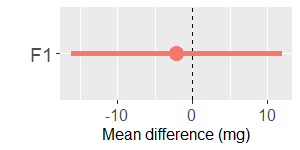 |
| Lungs | 214.10 (46.42) | 211.70 (47.20)  2.94  (-17.81, 23.53) | 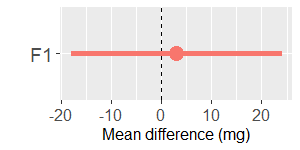 |
| Heart | 174.20 (30.72) | 167.40 (34.72)  3.34  (-4.09, 10.54) | 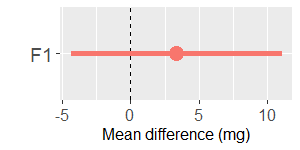 |
| Thymus | 11.52 (3.11) | 19.45 (33.93)  7.65  (-2.16, 17.46) | 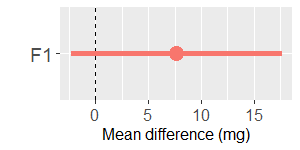 |
| Brain | 389.40 (24.21) | 387.40 (21.07)  0.86  (-10.12, 11.83) | 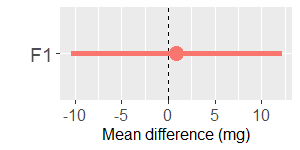 |

**Fig. S3. Weight of organs taken at 18 months of age (end of experiment).** Mean (SD) and Modelled mean difference control vs SSCT 95% CI. ^a^ denotes significant differences between control and SSCT.

**
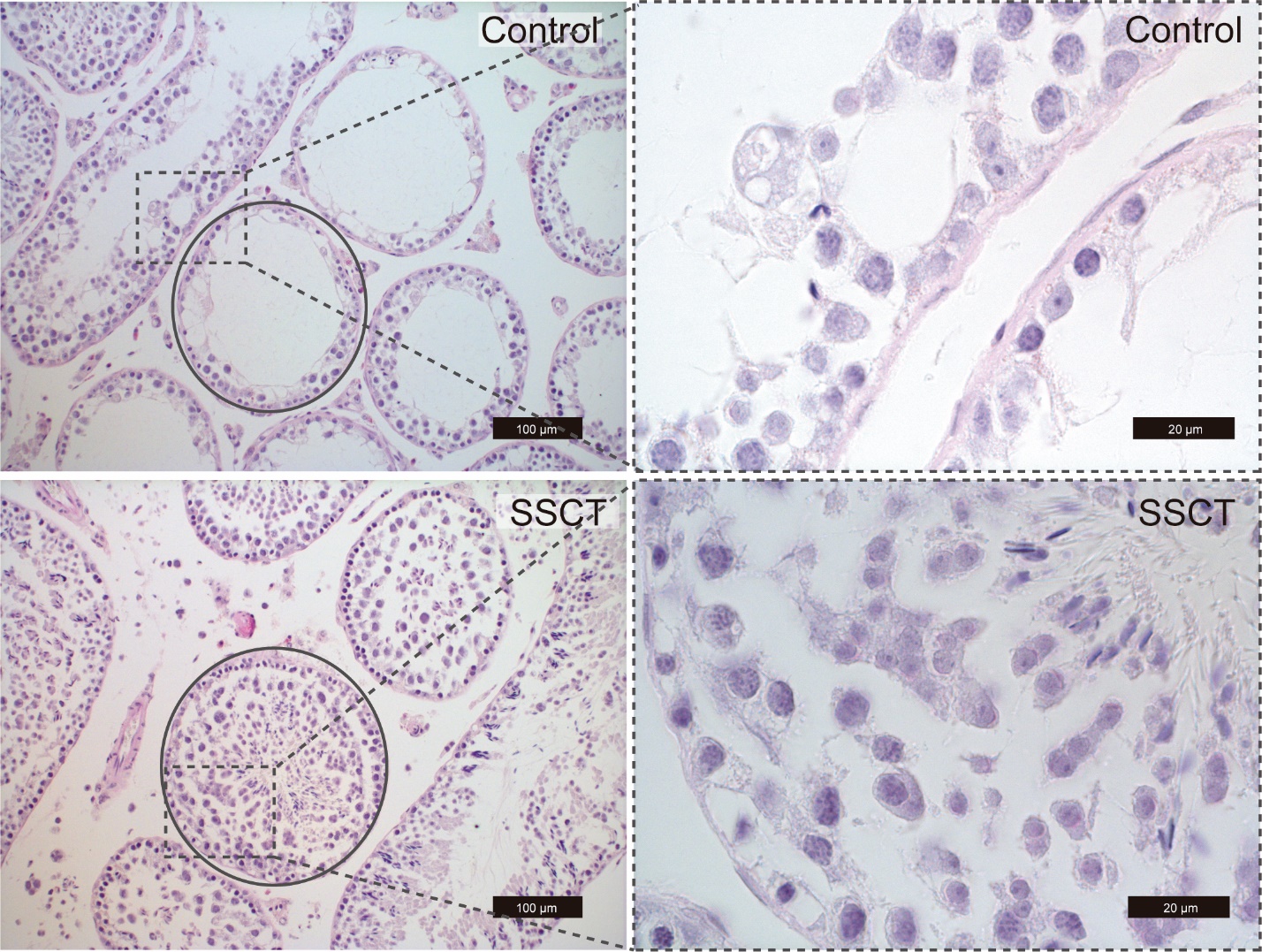
Fig. S4. Differences between control and SSCT seminiferous tubules.** Representative cross sections from the testis of control and SSCT mice used for calculating the tubular diameter of round seminiferous tubules of F1 control and SSCT mice. Round tubules are encircled by black dotted line. Scale bar, 100μm. Size is indicated in the scale bar.

**Table S1. Descriptive statistics of male and female developmental outcomes.** SSCT offspring compared to control, in both generations. Mean (SD).

| Outcome | Sex | | Control | F1 SSCT | F2 SSCT |
| --- | --- | --- | --- | --- | --- |
| Birthweight (g) | Male | | 1.35 (0.20) | 1.48 (0.29) | 1.28 (0.17) |
|  | Female | | 1.27 (0.18) | 1.30 (0.22) | 1.19 (0.16) |
| Birth length (cm) | Male | | 28.91 (1.86) | 30.16 (2.89) | 28.49 (1.) |
|  | Female | | 28.17 (1.53) | 28.79 (2.51) | 28.20 (1.24) |
| Full Fur growth (day) | Male | | 10.68 (0.77) | 10.74 (1.34) | 10.70 (1.13) |
|  | Female | | 10.74 (0.86) | 11.22 (1.47) | 10.62 (1.21) |
| Ear opening (day) | Male | | 13.00 (1.01) | 13.09 (1.44) | 13.68 (1.14) |
|  | Female | | 13.00 (1.14) | 13.45 (1.32) | 13.45 (1.25) |
| Eye opening (day) | Male | | 13.09 (0.88) | 12.79 (1.54) | 13.47 (0.86) |
|  | Female | | 13.26 (0.96) | 12.93 (1.35) | 13.49 (0.93) |
| Upper incisor (day) | Male | | 12.91 (1.95) | 12.32 (2.28) | 11.34 (0.88) |
|  | Female | | 12.75 (2.00) | 12.49 (2.18) | 11.53 (0.89) |
| Lower incisor (day) | Male | | 9.29 (0.95) | 8.69 (1.01) | 8.75 (0.84) |
|  | Female | | 9.24 (1.01) | 8.96 (1.03) | 9.14 (0.95) |
| Turn reflex (day) | Male | | 7.57 (1.36) | 7.31 (1.19) | 7.60 (1.32) |
|  | Female | | 7.71 (1.57) | 7.77 (1.12) | 8.06 (1.38) |
| Grasp reflex (day) | Male | | 12.82 (1.61) | 12.57 (2.46) | 13.32 (2.83) |
|  | Female | | 12.85 (1.61) | 12.72 (1.97) | 12.92 (2.07) |
| Geotaxis (day) | Male | | 14.47 (2.50) | 13.70 (2.20) | 14.46 (1.88) |
|  | Female | | 14.43 (2.71) | 14.35 (2.10) | 14.55 (2.49) |
| weight GTT (day) | | Male | 33.97 (4.58) | 32.67 (5.52) | NA |
|  |  | Female | 25.16 (3.67) | 25.76 (2.18) | NA |
| Fasting glucose (day) | | Male | 8.78 (1.72) | 8.81 (1.30) | NA |
|  |  | Female | 7.26 (0.97) | 7.21 (0.88) | NA |
| BP diastolic (mmHg) | | Male | 104.08 (15.58) | 103.80 (15.55) | NA |
|  |  | Female | 102.60 (9.52) | 103.20 (10.51) | NA |
| BP systolic (mmHg) | | Male | 143.00 (18.05) | 137.30 (17.02) | NA |
|  |  | Female | 132.40 (13.14) | 132.80 (11.80) | NA |
| Heart rate (bpm) | | Male | 548.90 (46.09) | 569.50 (26.29) | NA |
|  |  | Female | 582.40 (37.46) | 570.70 (39.98) | NA |
| Ovary R (mg) | | Female | 26.04 (7.54) | 19.96 (5.27) | NA |
| Ovary L (mg) | | Female | 39.26 (53.24) | 21.63 (5.48) | NA |
| Uterus (mg) | | Female | 307.60 (112.57) | 319.40 (81.64) | NA |
| Fat pad R (mg) | | Female | 925.90 (345.46) | 706.50 (399.31) | NA |
|  |  | Male | 607.40 (240.40) | 839.20 (381.01) | NA |
| Fat pad L (mg) | | Female | 834.30 (301.23) | 681.70 (381.01) | NA |
|  |  | Male | 554.60 (222.45) | 782.10 (355.92) | NA |
| Testis R (mg) | | Male | 81.21 (22.03) | 101.50 (22.79) | NA |
| Epididymis R (mg) | | Male | 21.72 (7.81) | 26.97 (6.77) | NA |
| Testis L (mg) | | Male | 72.63 (17.57) | 96.85 (18.76) | NA |
| Epididymis L (mg) | | Male | 22.19 (6.34) | 24.05 (3.81) | NA |
| Bladder (mg) | | Male | 25.78 (5.83) | 24.92 (6.19) | NA |
|  |  | Female | 24.09 (3.77) | 22.12 (3.36) | NA |
| Pancreas (mg) | | Male | 416.00 (60.40) | 492.30 (126.29) | NA |
|  |  | Female | 396.90 (42.91) | 389.10 (61.58) | NA |
| Intestines (mg) | | Male | 1190.00 (161.22) | 1114.00 (147.46) | NA |
|  |  | Female | 1165.00 (156.50) | 1115.00 (156.04) | NA |
| Spleen (mg) | | Male | 223.10 (452.35) | 248.80 (581.10) | NA |
|  |  | Female | 130.20 (76.70) | 97.12 (25.38) | NA |
| Stomach (mg) | | Male | 307.70 (62.71) | 261.60 (46.29) | NA |
|  |  | Female | 259.00 (32.53) | 239.20 (27.22) | NA |
| Liver (mg) | | Male | 2275.00 (672.79) | 1930.00 (422.44) | NA |
|  |  | Female | 1500.00 (172.45) | 1346.00 (215.27) | NA |
| Kidney R (mg) | | Male | 317.20 (32.52) | 299.90 (40.49) | NA |
|  |  | Female | 178.90 (38.22) | 169.60 (17.85) | NA |
| Kidney L (mg) | | Male | 305.80 (54.60) | 300.70 (40.69) | NA |
|  |  | Female | 184.80 (23.81) | 168.40 (24.86) | NA |
| Lungs (mg) | | Male | 220.00 (52.05) | 210.10 (40.84) | NA |
|  |  | Female | 206.30 (37.56) | 212.90 (52.15) | NA |
| Ventricle (mg) | | Male | 196.50 (20.71) | 198.30 (27.74) | NA |
|  |  | Female | 146.20 (12.98) | 144.80 (17.68) | NA |
| Thymus (mg) | | Male | 11.57 (3.35) | 18.26 (26.86) | NA |
|  |  | Female | 11.45 (2.82) | 20.36 (38.98) | NA |
| Brain (mg) | | Male | 388.60 (23.55) | 393.40 (18.13) | NA |
|  |  | Female | 390.50 (25.59) | 383.00 (22.31) | NA |

**Table S2. Observations in animals that died prematurely.** When possible, a necropsy was performed on animals that died before 18 months of age or had welfare issues.

| ID Code | Group | Gen. | Date of birth | Sex | Age at death (months) | Date of death | Observations |
| --- | --- | --- | --- | --- | --- | --- | --- |
| 7TUGL | SSCT | F1 | 13-7-2017 | F | 17 | 21-12-2018 | Sacrificed: severely enlarged abdomen |
| WT1NL | Control | F1 | 11-8-2017 | M | 10 | 18-6-2018 | Sacrificed; wounds because of fighting |
| 6D0AG | Control | F1 | 11-8-2017 | M | 10 | 21-6-2018 | Sacrificed; wounds because of fighting |
| 2YVLH | Control | F1 | 13-8-2017 | M | 17 | 2-1-2019 | Sacrificed; tail necrotic (2cm) |
| SLEE1 | Control | F1 | 13-8-2017 | M | 17 | 2-1-2019 | Sacrificed; thickened hind paws with wounds |
| XUOUV | SSCT | F1 | 17-10-2017 | M | 8 | 5-6-2018 | Sacrificed because of fighting wounds and red penis |
| 5MJ40 | SSCT | F1 | 21-10-2017 | M | 7 | 18-5-2018 | Sacrificed because of wound at penis |
| DWZOY | Control | F1 | 4-11-2017 | M | 17 | 12-4-2019 | Sacrificed because of red, bold scrotum |
| KJ1KL | Control | F1 | 15-11-2017 | M | 15 | 5-2-2019 | Sacrificed; wounds hind paws and red eye |
| ZBJX1 | Control | F1 | 15-11-2017 | M | 16 | 5-3-2019 | Found dead in cage |
| T1BGY | SSCT | F1 | 24-11-2017 | F | 10 | 18-9-2018 | Found dead in cage |
| CK39E | Control | F1 | 25-11-2017 | M | 13 | 17-12-2018 | Sacrificed by animal caretakers; large wounds (cadaver discarded by accident) |
| 8EIT8 | Control | F1 | 25-11-2017 | F | 15 | 15-2-2019 | Found dead in cage |
| Z8R71 | Control | F1 | 25-12-2017 | M | 15 | 2-4-2019 | Found dead in cage |
| DJBGO | SSCT | F1 | 9-1-2018 | M | 5 | 4-6-2018 | Sacrificed because of wounds to tail and paws. |
| NYP5W | SSCT | F1 | 9-1-2018 | F | 16 | 21-5-2019 | Sacrificed because of skin wound. |
| VF57Q | Control | F1 | 27-1-2018 | M | 10 | 4-12-2018 | Found dead in cage |
| QYC7L | SSCT | F1 | 30-1-2018 | M | 10 | 29-11-2018 | Bump in the abdomen with pus. Subcutaneous infection |
| HIEWO | Control | F1 | 8-2-2018 | F | 7 | 5-9-2018 | Found dead in cage |
| ON7M5 | Control | F1 | 8-2-2018 | F | 17 | 25-6-2019 | Found dead in cage |
| - | SSCT | F2 | 14-2-2018 | M | 1 | 13-3-2018 | Sacrificed due to teeth malformation and low weight |
| - | SSCT | F2 | 21-6-2018 | M | 3 | 21-9-2018 | Found dead in cage |

**Table S3.** **Final number of animals used for each assessment by sex, characteristic and generation.**

| Birth Assessment | Sex | | Control | | F1 SSCT | | F2 SSCT | | |
| --- | --- | --- | --- | --- | --- | --- | --- | --- | --- |
| Birth weight | Male | | 57 | | 32 | | 37 | | |
|  | Female | | 41 | | 43 | | 48 | | |
| Birth length | Male | | 57 | | 32 | | 37 | | |
|  | Female | | 41 | | 43 | | 49 | | |
| Childhood Assessment | **Sex** | **Control** | | **F1 SSCT** | | | **F2 SSCT** |  |  |
| Fur growth | Male | 56 | | 31 | | | 37 |  |  |
|  | Female | 42 | | 41 | | | 53 |  |  |
| Ear opening | Male | 56 | | 33 | | | 38 |  |  |
|  | Female | 41 | | 40 | | | 53 |  |  |
| Eye opening | Male | 56 | | 33 | | | 38 |  |  |
|  | Female | 42 | | 40 | | | 53 |  |  |
| Upper incisor | Male | 54 | | 28 | | | 38 |  |  |
|  | Female | 40 | | 37 | | | 53 |  |  |
| Lower incisor | Male | 56 | | 36 | | | 36 |  |  |
|  | Female | 42 | | 44 | | | 52 |  |  |
| Turn reflex | Male | 56 | | 36 | | | 37 |  |  |
|  | Female | 42 | | 44 | | | 53 |  |  |
| Grasp reflex | Male | 55 | | 30 | | | 37 |  |  |
|  | Female | 39 | | 36 | | | 51 |  |  |
| Geotaxis | Male | 47 | | 30 | | | 37 |  |  |
|  | Female | 40 | | 34 | | | 53 |  |  |
| Adulthood Assessment | **Sex** | **Control** | | | | **F1 SSCT** | **F2 SSCT** | |  |
| Weight GTT | Male | 10 | | | | 10 | NA | |  |
|  | Female | 10 | | | | 10 | NA | |  |
| Fasting glucose | Male | 10 | | | | 10 | NA | |  |
|  | Female | 10 | | | | 10 | NA | |  |
| BP diastolic | Male | 12 | | | | 13 | NA | |  |
|  | Female | 13 | | | | 13 | NA | |  |
| BP systolic | Male | 12 | | | | 13 | NA | |  |
|  | Female | 13 | | | | 13 | NA | |  |
| Heart rate | Male | 12 | | | | 13 | NA | |  |
|  | Female | 13 | | | | 13 | NA | |  |
| Ovary R | Male | 0 | | | | 0 | NA | |  |
|  | Female | 23 | | | | 27 | NA | |  |
| Ovary L | Male | 0 | | | | 0 | NA | |  |
|  | Female | 23 | | | | 27 | NA | |  |
| Uterus | Male | 0 | | | | 0 | NA | |  |
|  | Female | 22 | | | | 27 | NA | |  |
| Fat pad R | Male | 29 | | | | 20 | NA | |  |
|  | Female | 23 | | | | 27 | NA | |  |
| Fat pad L | Male | 30 | | | | 18 | NA | |  |
|  | Female | 23 | | | | 27 | NA | |  |
| Testis R | Male | 30 | | | | 20 | NA | |  |
|  | Female | 0 | | | | 0 | NA | |  |
| Epididymis R | Male | 29 | | | | 19 | NA | |  |
|  | Female | 0 | | | | 0 | NA | |  |
| Testis L | Male | 31 | | | | 20 | NA | |  |
|  | Female | 0 | | | | 0 | NA | |  |
| Epididymis L | Male | 31 | | | | 19 | NA | |  |
|  | Female | 0 | | | | 0 | NA | |  |
| Bladder | Male | 31 | | | | 19 | NA | |  |
|  | Female | 22 | | | | 26 | NA | |  |
| Pancreas | Male | 29 | | | | 18 | NA | |  |
|  | Female | 23 | | | | 26 | NA | |  |
| Intestines | Male | 28 | | | | 20 | NA | |  |
|  | Female | 23 | | | | 27 | NA | |  |
| Spleen | Male | 27 | | | | 18 | NA | |  |
|  | Female | 23 | | | | 24 | NA | |  |
| Stomach | Male | 31 | | | | 19 | NA | |  |
|  | Female | 23 | | | | 26 | NA | |  |
| Liver | Male | 30 | | | | 20 | NA | |  |
|  | Female | 23 | | | | 26 | NA | |  |
| Kidney R | Male | 28 | | | | 19 | NA | |  |
|  | Female | 22 | | | | 26 | NA | |  |
| Kidney L | Male | 28 | | | | 19 | NA | |  |
|  | Female | 22 | | | | 26 | NA | |  |
| Lungs | Male | 30 | | | | 20 | NA | |  |
|  | Female | 23 | | | | 27 | NA | |  |
| Heart  Ventricle | Male | 29 | | | | 19 | NA | |  |
|  | Female | 23 | | | | 26 | NA | |  |
| Thymus | Male | 28 | | | | 19 | NA | |  |
|  | Female | 20 | | | | 25 | NA | |  |
| Brain | Male | 30 | | | | 20 | NA | |  |
|  | Female | 22 | | | | 27 | NA | |  |

**Table S4.** **Sample size and power calculations**. One sided, calculated in Stata IC 14.2, with continuity.

| Test | N female | N male | Age | References for expected difference between groups |
| --- | --- | --- | --- | --- |
| **Congenital abnormalities** | 58 | 58 | 0 days | [15, 17, 20] |
| **Birthweight** | 19 | 19 | 0 days | [21] |
| **Righting reflex** | 29 | 29 | 0-28 days | [6] |
| **Blood pressure** | 13 | 13 | 8 months | [22] |
| **Glucose tolerance test** | 10 | 10 | 9-12 months | [21] |

**Movie legends**

**Movie S1.** Righting reflex level 1. The animal lies on its back, moving its paws in the air without any direction and there is no righting.

**Movie S2.** Righting reflex level 2. The animal rights itself, but slowly.

**Movie S3.** Righting reflex level. The animal rights itself, but slowly.

**Movie S4.** Grasping reflex level 1. The animal puts its paw on the cotton bud, but it does not hold.

**Movie S5.** Grasping reflex level 2. The object is firmly grasped and the mouse it is able to grasp and hold the stick.

**Movie S6.** Negative geotaxis level. The animal turns its body slowly on the slope but stops halfway without moving any further.

**Movie S7.** Negative geotaxis level 2. The animal turns its body, and then goes up the ramp but stops halfway.

**Movie S8.** Negative geotaxis level 3. The animal turns by 180° and moves towards the top of the slope immediately reaching the top.

**References**

1. Mulder CL, Catsburg LAE, Zheng Y, et al (2018) Long-term health in recipients of transplanted in vitro propagated spermatogonial stem cells. Hum Reprod 33:81–90. https://doi.org/10.1093/humrep/dex348

2. Kanatsu-Shinohara M, Ogonuki N, Inoue K, et al (2003) Long-term proliferation in culture and germline transmission of mouse male germline stem cells. Biol Reprod 69:612–6. https://doi.org/10.1095/biolreprod.103.017012

3. Mulder CL, Serrano JB, Catsburg LAE, et al (2018) A practical blueprint to systematically study life-long health consequences of novel medically assisted reproductive treatments. Hum Reprod 33:784–792. https://doi.org/10.1093/humrep/dey070

4. Kanatsu-Shinohara M, Ogonuki N, Inoue K, et al (2003) Long-Term Proliferation in Culture and Germline Transmission of Mouse Male Germline Stem Cells. Biol Reprod 69:612–616. https://doi.org/10.1095/biolreprod.103.017012

5. Mulder CL, Catsburg LAE, Zheng Y, et al (2018) Long-term health in recipients of transplanted in vitro propagated spermatogonial stem cells. Hum Reprod 33:81–90. https://doi.org/10.1093/humrep/dex348

6. Tarín JJ, Pérez-Albalá S, Aguilar a, et al (1999) Long-term effects of postovulatory aging of mouse oocytes on offspring: a two-generational study. Biol Reprod 61:1347–1355. https://doi.org/10.1095/biolreprod61.5.1347

7. van der Meer M, Costa P, Baumans V, et al (1999) Welfare assessment of transgenic animals: Behavioural responses and morphological development of newborn mice. Altern To Lab Anim 27:857–868

8. van der Meer M, Baumans V, Hofhuis FMA, et al (2001) Consequences of gene targeting procedures for behavioural responses and morphological development of newborn mice. Transgenic Res 10:399–408. https://doi.org/10.1023/A:1012244404020

9. Silver LM (1995) Mouse Genetics: Concepts and Applications. Oxford Press University

10. van der Meer M, Baumans V, Hofhuis FMA, et al (2001) Consequences of gene targeting procedures for behavioural responses and morphological development of newborn mice. Transgenic Res 10:399–408. https://doi.org/10.1023/A:1012244404020

11. Ayala JE, Samuel VT, Morton GJ, et al (2010) Standard operating procedures for describing and performing metabolic tests of glucose homeostasis in mice. DMM Dis Model Mech 3:525–534. https://doi.org/10.1242/dmm.006239

12. Andrikopoulos S, Blair AR, Deluca N, et al (2008) Evaluating the glucose tolerance test in mice. Am J Physiol - Endocrinol Metab 295:1323–1332. https://doi.org/10.1152/ajpendo.90617.2008

13. Wang Y, Thatcher SE, Cassis LA (2017) Measuring blood pressure using a noninvasive tail cuff method in mice. Methods Mol Biol 1614:69–73. https://doi.org/10.1007/978-1-4939-7030-8_6

14. Jørgensen MS, Tornqvist KS, Hvid H (2017) Calculation of Glucose Dose for Intraperitoneal Glucose Tolerance Tests in Lean and Obese Mice. 56:95–97

15. Hansen M, Kurinczuk JJ, Milne E, et al (2013) Assisted reproductive technology and birth defects: a systematic review and meta-analysis. Hum Reprod Update 19:330–353. https://doi.org/10.1093/humupd/dmt006

16. Davies MJ, Moore VM, Willson KJ, et al (2012) Reproductive technologies and the risk of birth defects. N Engl J Med 366:1803–13. https://doi.org/10.1056/NEJMoa1008095

17. Alukal JP, Lipshultz LI (2008) Safety of assisted reproduction, assessed by risk of abnormalities in children born after use of in vitro fertilization techniques. Nat Clin Pr Urol 5:140–150. https://doi.org/10.1038/ncpuro1045

18. Althouse AD (2016) Adjust for Multiple Comparisons? It’s Not That Simple. Ann Thorac Surg 101:1644–1645. https://doi.org/10.1016/j.athoracsur.2015.11.024

19. Bates D, Mächler M, Bolker BM, Walker SC (2015) Fitting linear mixed-effects models using lme4. J Stat Softw 67:1–48. https://doi.org/10.18637/jss.v067.i01

20. Davies MJ, Moore VM, Willson KJ, et al (2012) Reproductive Technologies and the Risk of Birth Defects. N Engl J Med 366:1803–1813. https://doi.org/10.1056/NEJMoa1008095

21. Chen M, Wu L, Zhao J, et al (2014) Altered glucose metabolism in mouse and humans conceived by IVF. Diabetes 63:3189–3198. https://doi.org/10.2337/db14-0103

22. Watkins AJ, Platt D, Papenbrock T, et al (2007) Mouse embryo culture induces changes in postnatal phenotype including raised systolic blood pressure. Proc Natl Acad Sci U S A 104:5449–54. https://doi.org/10.1073/pnas.0610317104
